# Advanced stage, high-grade primary tumor ovarian cancer: a multi-omics dissection and biomarker prediction process

**DOI:** 10.1038/s41598-023-44246-9

**Published:** 2023-10-12

**Authors:** Yousof Saeedi Honar, Saleh Javaher, Marziye Soleimani, Amir Zarebkohan, Behrouz Farhadihosseinabadi, Masoud Tohidfar, Meghdad Abdollahpour-Alitappeh

**Affiliations:** 1 Department of Plant Biotechnology, Faculty of Life Sciences and Biotechnology, Shahid Beheshti University, Tehran, 1983963113 Iran; 2 Department of Cell and Molecular Biology, Faculty of Life Sciences and Biotechnology, Shahid Beheshti University, Tehran, 1983969411 Iran; 3 Department of Medical Nanotechnology, Drug Applied Research Center, Faculty of Advanced Medical Sciences, Tabriz University of Medical Sciences, Tabriz, 516661-4733 Iran; 4 Hematopoietic Stem Cell Research Center, Shahid Beheshti University of Medical Sciences, Tehran, Iran; 5 Cellular and Molecular Biology Research Center, Larestan University of Medical Sciences, Larestan, Iran

**Keywords:** Cancer, Biomarkers, Health care, Risk factors

## Abstract

Ovarian cancer (OC) incidence and mortality rates continue to escalate globally. Early detection of OC is challenging due to extensive metastases and the ambiguity of biomarkers in advanced High-Grade Primary Tumors (HGPTs). In the present study, we conducted an in-depth in silico analysis in OC cell lines using the Gene Expression Omnibus (GEO) microarray dataset with 53 HGPT and 10 normal samples. Differentially-Expressed Genes (DEGs) were also identified by GEO2r. A variety of analyses, including gene set enrichment analysis (GSEA), ChIP enrichment analysis (ChEA), eXpression2Kinases (X2K) and Human Protein Atlas (HPA), elucidated signaling pathways, transcription factors (TFs), kinases, and proteome, respectively. Protein–Protein Interaction (PPI) networks were generated using STRING and Cytoscape, in which co-expression and hub genes were pinpointed by the cytoHubba plug-in. Validity of DEG analysis was achieved via Gene Expression Profiling Interactive Analysis (GEPIA). Of note, KIAA0101, RAD51AP1, FAM83D, CEP55, PRC1, CKS2, CDCA5, NUSAP1, ECT2, and TRIP13 were found as top 10 hub genes; SIN3A, VDR, TCF7L2, NFYA, and FOXM1 were detected as predominant TFs in HGPTs; CEP55, PRC1, CKS2, CDCA5, and NUSAP1 were identified as potential biomarkers from hub gene clustering. Further analysis indicated hsa-miR-215-5p, hsa-miR-193b-3p, and hsa-miR-192-5p as key miRNAs targeting HGPT genes. Collectively, our findings spotlighted HGPT-associated genes, TFs, miRNAs, and pathways as prospective biomarkers, offering new avenues for OC diagnostic and therapeutic approaches.

## Introduction

Ovarian cancer (OC) ranks as the fifth leading cause of cancer-related deaths globally, resulting in significant mortality among women. OC is projected to increase mortality by 2035, mainly due to its increasing burden in low- and middle-income countries. The World Health Organization (WHO) has categorized OC into five distinct histological subtypes, including high-grade serous carcinoma (HGSC), low-grade serous carcinoma (LGSC), mucinous carcinoma (MC), endometrioid carcinoma (EC) and clear cell carcinoma (CCC). Of note, each subtype is characterized by its unique risk factors, cellular origins, molecular compositions, clinical presentations, and therapeutic approaches^1^. Despite advances, the persistently high incidence and mortality rates of OC over the past two decades can be attributed to the limited efficacy of existing therapies in prolonging overall survival (OS) beyond five years for advanced-stage patients and challenges in early and effective diagnosis. Early diagnosis of OC, due to extensive metastases and the lack of biomarkers in advanced stages of high-grade primary tumors (HGPTs), remains one of the most important challenges^2,3^.

Over the past decade, the oncology field has increasingly prioritized rapid, reliable, and precise cancer detection methods. Numerous approaches have emerged for the ﻿discovery of biomarkers that play a pivotal role in early cancer detection. Molecular profiling, for example, offers promising strategies for the diagnosis of patients with OC. Of note, multi-omics data provide a comprehensive understanding of tumor biology, paving the way for the identification of prognostic biomarkers. Such biomarkers have the potential to significantly enhance early diagnosis and prognostic prediction for aggressive OCs, in turn governing treatment outcomes. By exploring the hallmarks of OC, similar to other solid tumors, researchers can increase the likelihood of discovering potential biomarkers in the early stage of OC^4–6^.

Understanding of intrinsic signaling pathways, angiogenesis, hormone receptors, and immunologic factors involved in OC pathogenesis seems to be potential theranostic targets. Similar to other normal and malignant cells, OC cells have their own unique transcriptome, proteome, epigenome, and metabolome. Transcriptome analysis is typically used to characterize transcriptional activity (coding and non-coding RNAs), and provides a snapshot of actively-expressed genes and transcripts under diverse situations, such as cancer^7^. Bioinformatics, a science combining molecular biology and information technology, is being used to study the molecular mechanisms controlling normal and abnormal biological processes. Bioinformatics and computational models have been well used to study various tumors, demonstrating to be an efficient and reliable approach in the identification of novel tumor markers for cancer diagnosis and targeted therapies^8^. In recent decades, high-throughput technologies, such as microarray, have provided large expression data sets and discovered a large number of disease/tumor markers^9^. Such discoveries remarkably improved the early diagnosis and prognosis of tumors^10,11^. The microarray technology, in combination with bioinformatics analysis, has been used to analyze a variety of cancers^12,13^; Importantly, microarray was demonstrated to be an appropriate approach to comprehensively analyze the genes involved in the development and progression of OC^14^.

**Figure 1:**
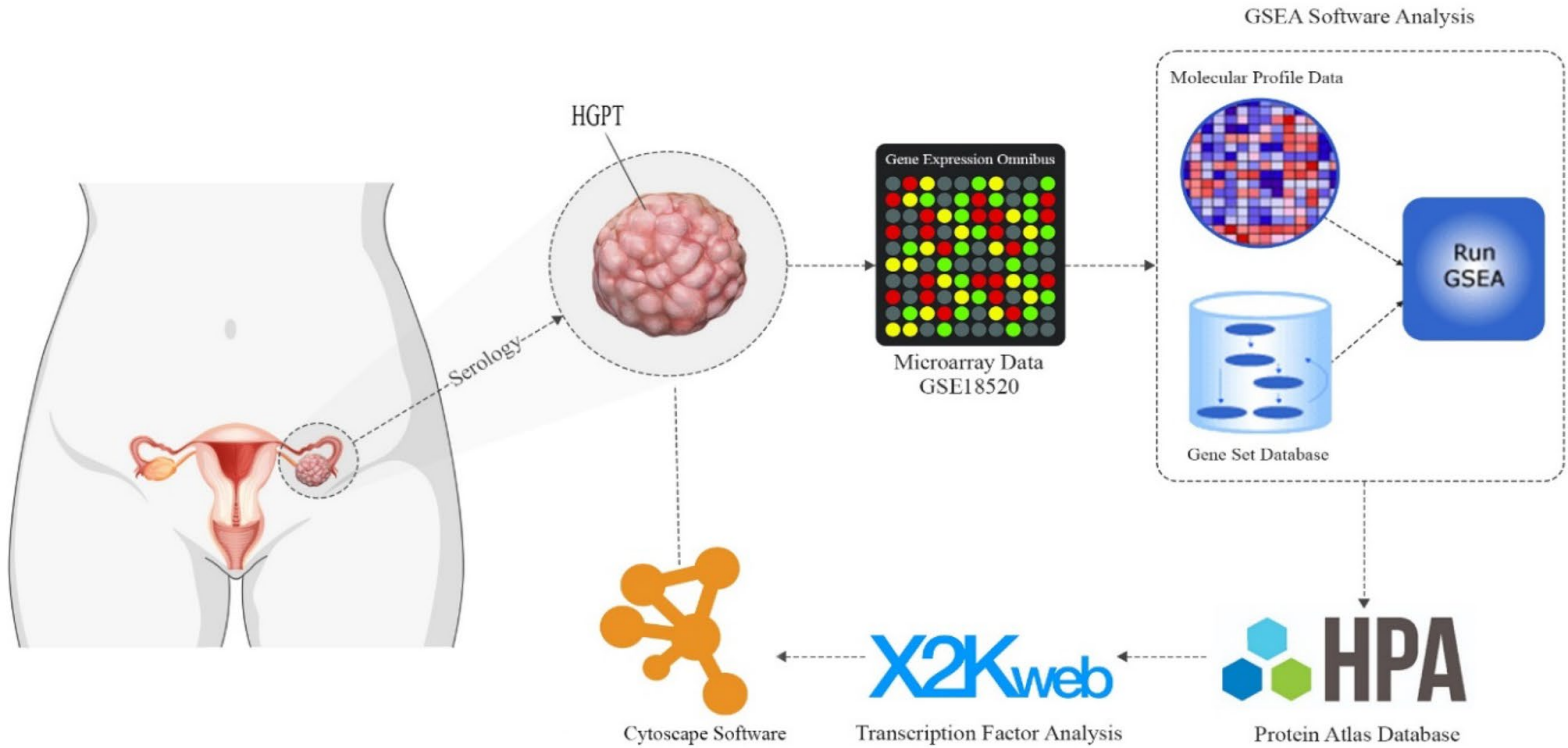
An overview of analyses carried out in this study.

In the present study, in silico approaches were conducted to leverage multi-omics data for OC biomarker prediction. We analyzed microarray data comparing HGPT to normal samples, identifying upregulated genes associated with HGPTs. These genes underwent a multifaceted analysis, including gene set enrichment analysis (GSEA), Kyoto Encyclopedia of Genes and Genomes (KEGG) pathway mapping, protein–protein interaction (PPI), co-expression profiling, biomarker clustering, and Human Protein Atlas (HPA) analysis (Fig. 1).

## Materials and methods

### Microarray data and gene expression profile analysis

Gene Expression Omnibus (GEO), a database for gene expression and RNA methylation profilings managed by the National Center for Biotechnology Information (NCBI), supports reporting standards derived from the scientific community; GEO determines the presence of several critical study elements including raw data, processed data, and descriptive metadata^15^. The whole-genome oligonucleotide expression analysis of papillary serous ovarian adenocarcinomas data was acquired from NCBI GEO database (http://www.ncbi.nlm.nih.gov/geo). The gene expression profile dataset with the access number GSE18520 was retrieved from GEO (GPL570 [HG-U133_Plus_2] Affymetrix Human Genome U133 Plus 2.0 Array). Samples with |Log FC|> 2 and *p* < 0.05 were screened and considered to be statistically significant; these samples included 53 HGPT and 10 normal ovarian surface epithelium (OSE) samples.

### Protein–protein interaction (PPI) network and co-expression analysis

The goal of the STRING database is to integrate all known and predicted associations between proteins, including both physical interactions and functional associations. To achieve this goal, STRING collects and evaluates evidence from several sources, including (i) automated text retrieving of the scientific literature, (ii) databases of interaction experiments and annotated complexes/pathways, (iii) computational interaction predictions from co-expression and preserved genomic context, and (iv) systematic transmission of interaction evidence from one organism to another^14^. STRING is the search tool for retrieval of interacting genes database (version 11.5; https://string-db.org) which integrates both known and predicted PPIs and predicts functional interactions between DEGs (high confidence score 0.700 was set as the cut-off criteria to construct the PPI network). Ultimately, the cytoHubba (version 0.1) plug-in of the Cytoscape software (version 3.9.1; www.cytoscape. org) was used to identify hub genes.

### Decoding biological significance: gene set enrichment analysis (GSEA)

The GSEA software (version 4.2.3, https://www.gsea-msigdb.org/gsea/index.jsp) was used to determine the enrichment of the dataset obtained from the expression matrix of the GPL570-GSE18520 datasets downloaded from the GEO database. GSEA was performed to compare the molecular profile data with the priori-defined gene sets available at Molecular Signatures DataBase (MolSigDB). The KEGG gene sets were employed for the detection of signaling pathways^16^.

### Identification of MicroRNA (miRNA)-Targeted Genes

Enrichr dataset-linked miRTarBase (http://amp.pharm.mssm.edu) was used to find the top 10 microRNAs (miRNAs) that presumably target HGPT-related genes. miRTarBase provides information about experimentally-validated miRNA-target interactions (MTIs), whose new updated version has accumulated more than 13,404 validated MTIs from 11,021 articles from manual curations^17^. Top 10 miRNAs targeting HGPT-related genes were selected and ranked based on *p*-value (*P* ≤ 0.05).

### Detection of Transcription Factors (TFs) and Kinases

The ChIP enrichment analysis (ChEA) database was used to find transcription factors (TFs), which potentially control the expression of HGPT-related genes. The ChEA database provides data on eukaryotic TFs, consensus bond sequences (positional weight matrices), experimentally proven bond regions, and regulated genes^18^. In addition, eXpression2Kinases (X2K) (https://amp.pharm.mssm.edu/X2K/) was used to identify and rank putative TFs, protein complexes, and protein kinases which are most likely responsible for the observed changes in HGPT transcriptomes.

### The possible role of long non-coding RNAs (lncRNAs) in HGPT

Long non-coding RNAs (lncRNAs) may regulate cell proliferation, apoptosis, migration, invasion and maintenance of stemness during cancer development^19^. Therefore, our ultimate goal was to demonstrate the relation between the lncRNAs and HGPT genes. To assess our targeted lncRNAs, we used lncHUB database analysis and trimmed our dataset based on *p*-value (*p* ≤ 0.05).

### Hub gene selection and validation in the human protein atlas (HPA)

The hub gene expression level between cancer patients and healthy controls were identified by using the HPA database (https://www.proteinatlas.org/), a Swedish-based program initiated in 2003 with the goal of surveying all the human proteins in cells, tissues, and organs using an integration of various omics technologies^20^. We also visualized the expression of key hub genes in HGPT samples and normal ovarian surface epithelia using boxplots and Gene Expression Profiling Interactive Analysis (GEPIA), a recently-developed interactive web server able to analyze RNA sequencing expression data of 9736 tumors, 8587 normal samples from the TCGA and the GTEx projects, by using a standard processing pipeline^21^.

## Results

### Identification of differentially-expressed genes (DEGs)

**Figure 2:**
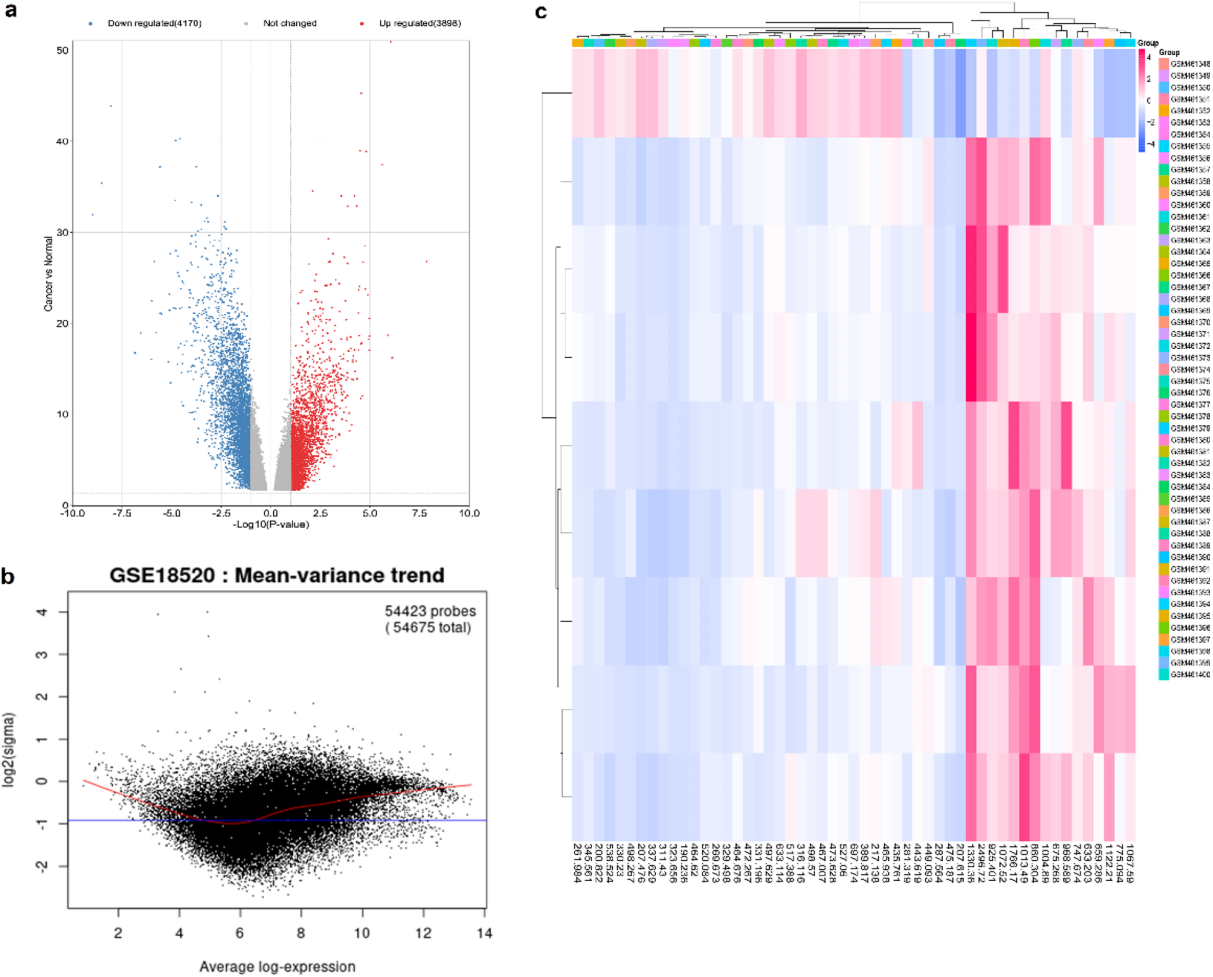
Gene expression analysis. (**a**) A volcano graphic illustrates data on differentially-expressed gene (DEG) down- and up-regulated genes colored by blue and red, respectively. (**b**) The voom plot illustrates the relationship between the coefficients of variation on the count size of significant genes. (**c**) The heatmap shows the expression level of hub genes in various samples.

**Figure 3:**
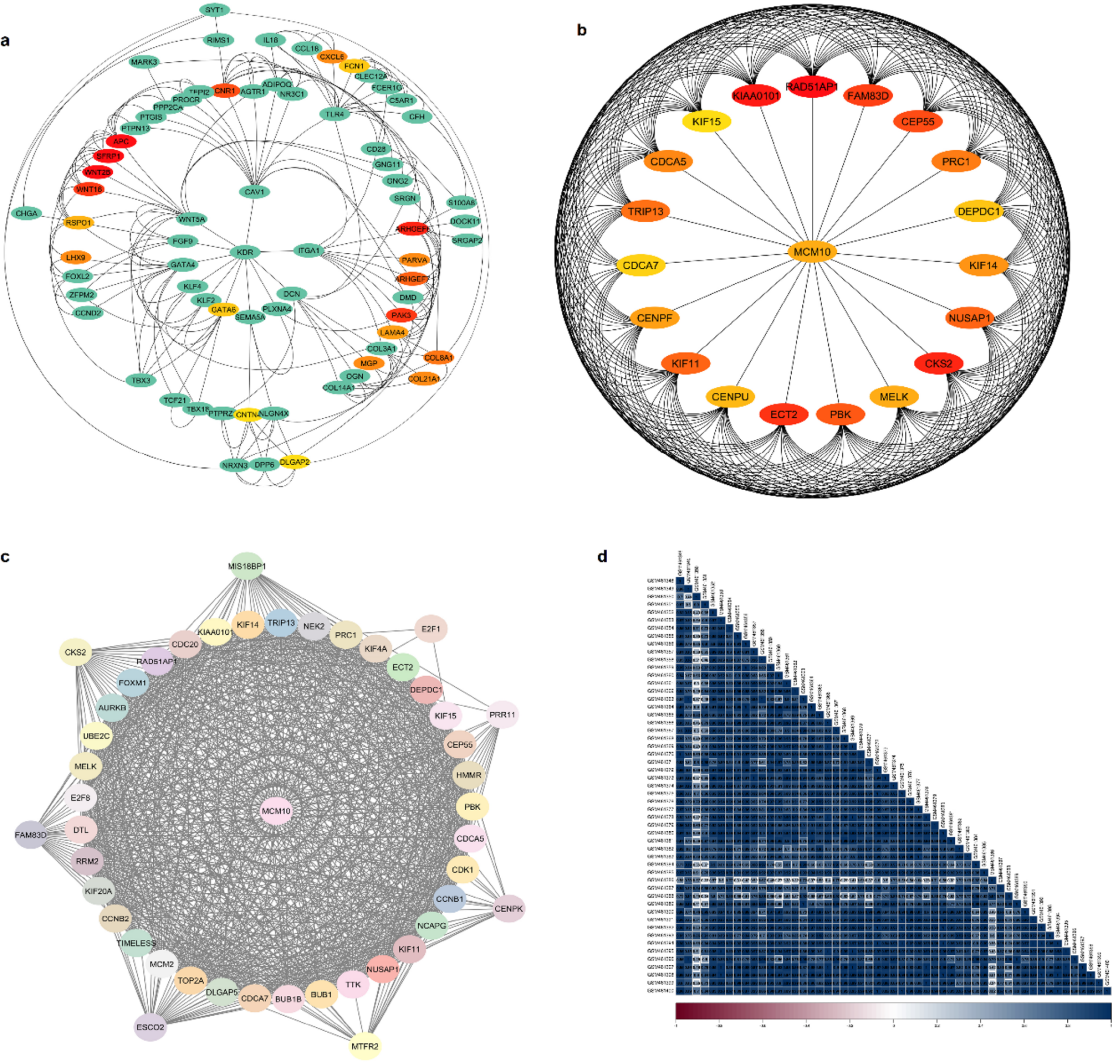
Protein–protein network analysis. (**a**) Protein–protein interaction (PPI) network of ovarian surface epithelium (OSE)-related genes. (**b**) PPI network of high-grade primary tumor (HGPT)-related genes. (**c** and **d**) co-expression analysis and graphical interaction between hub and non-target genes, as well as the construction of a correlation heatmap.

The differentially-expressed gene (DEG) up- and down-regulated genes were screened among the defined groups (53 HGPT and 10 normal ovarian surface epithelium samples). The Limma R packages were used to identify DEGs. *P*-value < 0.05 and |LogFC|> 2 were considered to be statistically significant and displayed using Volcano and Voom plots, showing that averages were log2-transformed mean-counts with a two-standard-deviation-offset (Fig. 2a,b). The interactions of up- and down-regulated genes were investigated using the STRING database. It was found that 642 and 917 genes were up- and down-regulated genes as HGPT-related and OSE-related genes, respectively; the expression level of these genes was displayed as the heatmap in all normal and cancer samples (Fig. 2c). Cytoscape software v 3.9.1 (cytoHubba plug-in) analysis was used to identify Hub genes (Fig. 3a,b). Additionally, approaches for gene co-expression analysis were carried out with the assistance of STRING database tools. In active interaction sources tools, we selected the co-expression analysis, followed by the minimum needed interaction score option with high confidence. According to the results, 46 genes from the hub gene list were potentially correlated to the co-expression network. The correlation value of genes was calculated using a correlation plot (Fig. 3c,d).

### Unraveling biological insights through gene set enrichment analysis (GSEA)

**Figure 4:**
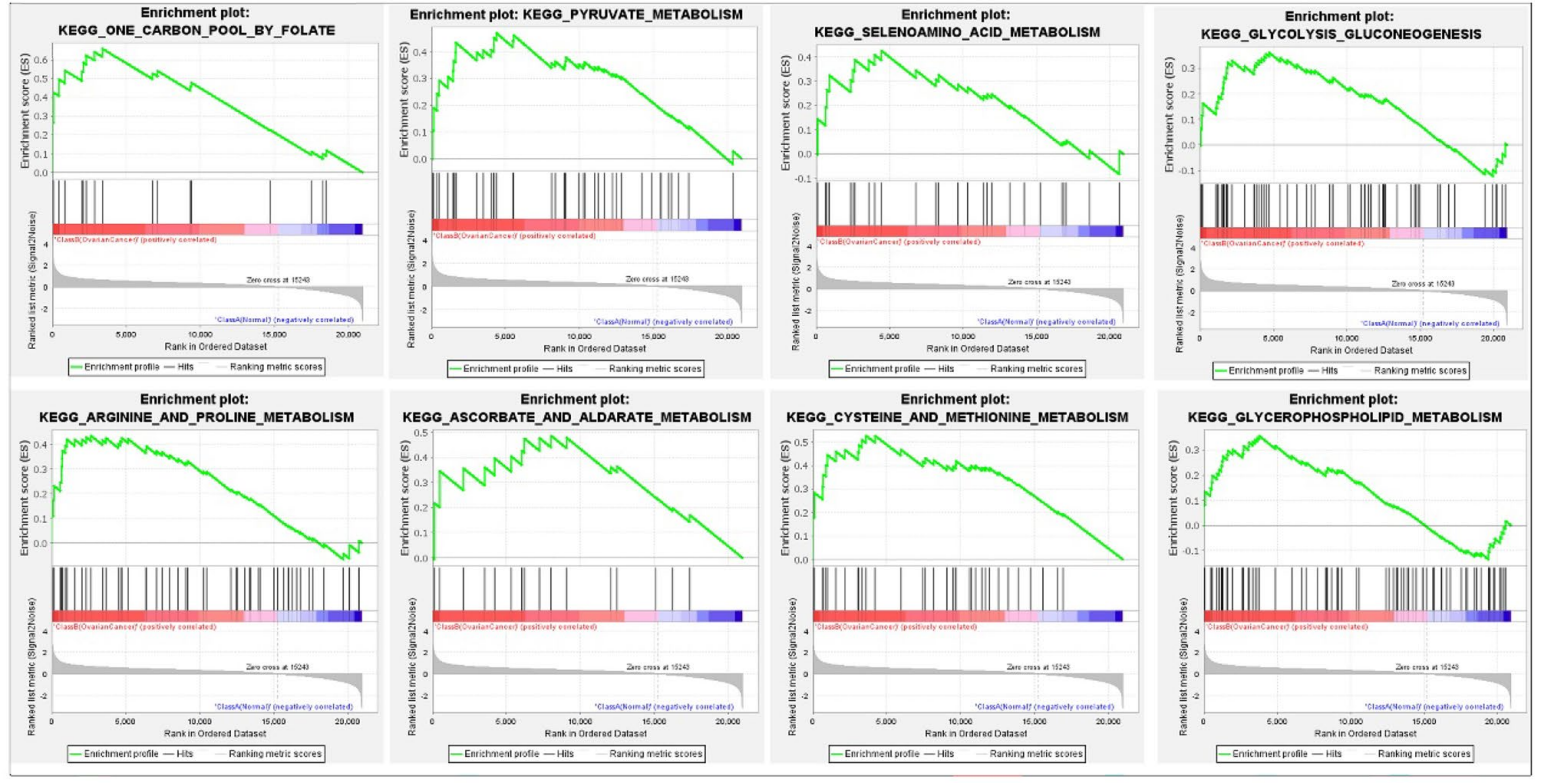
Pathway enrichment analysis and visualization of omics data using gene set enrichment analysis (GSEA) software. GSEA plots show the most enriched gene sets in metabolism pathways; twenty-four and 105 gene sets are significantly enriched at nominal *p*-value < 1% and *p*-value < 5%, respectively (permission has been obtained from Kanehisa laboratories from using KEGG pathway database^32–34^).

GSEA was performed using the KEGG package in the GSEA software environment for statistical analysis. Our results showed the expression of the genes in the data matrix targeting signaling pathways which are essential for the cell’s metabolic functions, including one-carbon pool by folate, pyruvate metabolism, selenoamino acid metabolism, glycolysis gluconeogenesis, arginine and proline metabolism, ascorbate and aldarate metabolism, cysteine and methionine metabolism, and glycerophospholipid metabolism (Fig. 4).

### MicroRNA target gene identification

ChEA, which is one of the Enrichr tools linked to miRTarBase, was used to identify the top 10 miRNAs to target HGPT-related genes. We found that three of the top 10 miRNAs, including hsa-miR-215-5p, hsa-miR-193b-3p and hsa-miR-192-5p that play a critical role in tumor suppression, have the most commonality with target genes (Table 1).

**Table 1:** Identification of the key miRNAs and genes involved in ovarian cancer.

Term	*P*-value	Target genes
Key miRNAs targeting up-regulated genes (ChEA)		
hsa-miR-215-5p	1.72E−08	CENPF; KIF14; DEPDC1; MCM10; TRIP13; FAM83D; ECT2; CEP55; KIF15
hsa-miR-193b-3p	4.87E−08	CENPU; MELK; CDCA5; CDCA7; MCM10; KIF11; TRIP13; ECT2; KIF15
hsa-miR-192-5p	1.81E−07	CENPF; KIF14; DEPDC1; MCM10; TRIP13; FAM83D; ECT2; CEP55; KIF15
hsa-miR-6507-5p	2.89E−04	PRC1; KIF11; CEP55
hsa-miR-4473	0.001416427	PRC1; DEPDC1
hsa-miR-373-3p	0.001696928	CENPF; MELK; PRC1; PBK
hsa-miR-4255	0.003079761	KIF14; ECT2
hsa-miR-340-5p	0.004479009	CDCA7; DEPDC1; KIF11
hsa-miR-493-5p	0.005819938	DEPDC1; CKS2
hsa-miR-18b-5p	0.005918504	RAD51AP1; CDCA5
mmu-miR-6951-3p	0.005985691	MCM10
mmu-miR-7116-3p	0.005985691	MCM10
mmu-miR-193a-3p	0.007973357	KIF15
mmu-miR-193b-3p	0.009957248	KIF15
hsa-miR-124-3p	0.012137728	RAD51AP1; CDCA7; DEPDC1; CKS2; FAM83D
Key miRNAs targeting down-regulated genes (ChEA)		
hsa-miR-24-2-5p	4.63E−04	PARVA; RSPO1
hsa-miR-24-1-5p	5.86E−04	PARVA; RSPO1
hsa-miR-1256	9.58E−04	WNT2B; ARHGEF7
hsa-miR-888-5p	0.002590114	PARVA; ARHGEF6
hsa-miR-6757-3p	0.00286498	WNT2B; GATA6
hsa-miR-135b-5p	0.003079761	APC; GATA6
hsa-miR-135a-5p	0.003929872	APC; GATA6
hsa-miR-7850-5p	0.0044345	WNT2B; GATA6
hsa-miR-6758-3p	0.005151575	RSPO1; PAK3
hsa-miR-6761-5p	0.005151575	GATA6; WNT16
hsa-miR-564	0.005433372	GATA6; COL8A1
mmu-miR-466i-3p	0.005582627	CXCL6; CNR1; COL8A1
hsa-miR-494-3p	0.008278453	CNR1; WNT16
mmu-miR-7b-5p	0.009014359	SFRP1; CNR1; PARVA
hsa-miR-429	0.009841197	GATA6; WNT16

### Identification of kinases and transcription factors (TFs)

**Figure 5:**
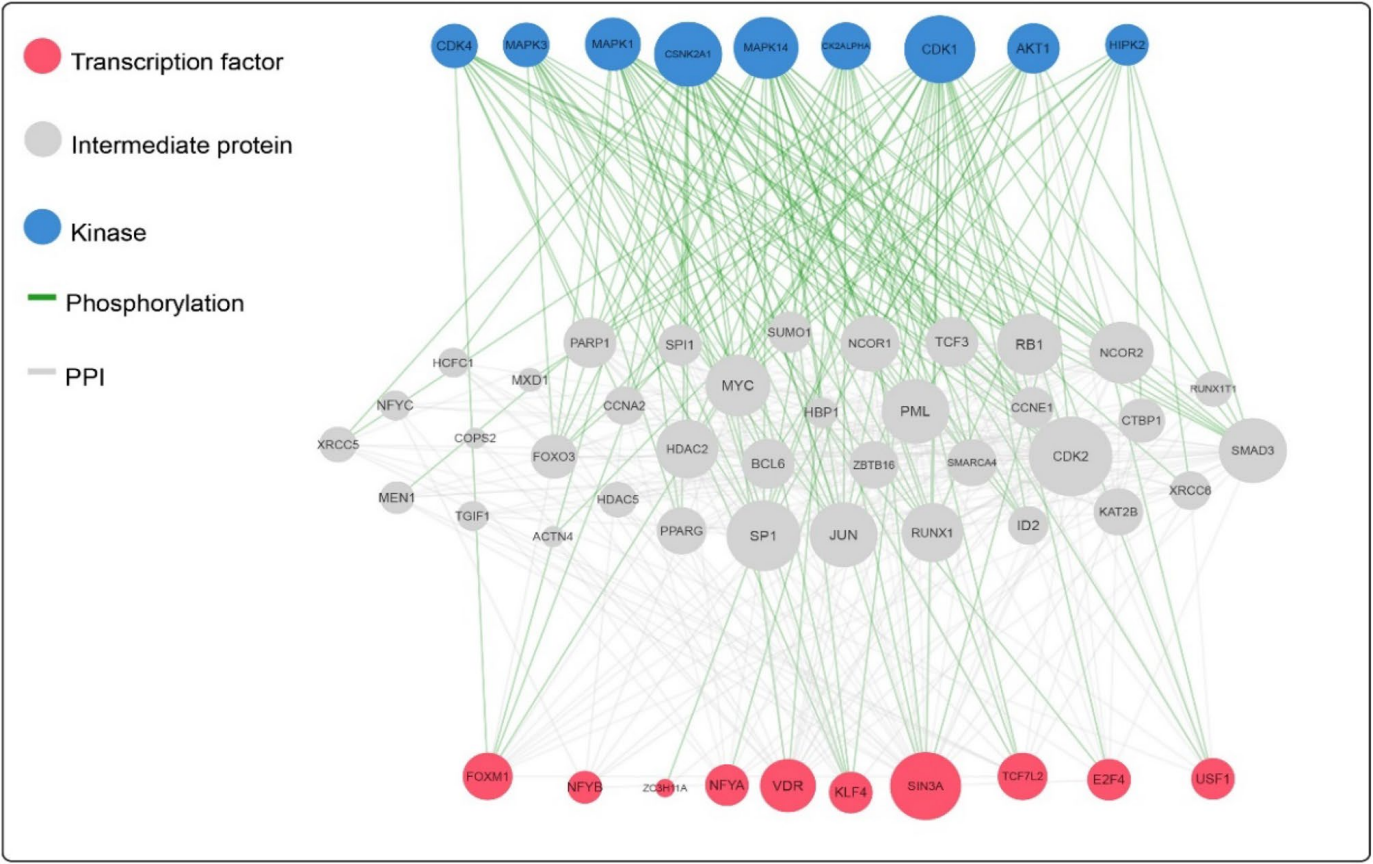
The interaction of transcription factors (TFs; red spots) and kinases (blue spots) with hub genes.

X2K was used to identify the key TFs, kinases, and intermediary proteins involved in the regulation of gene expression. Our results revealed that SIN3A, VDR, FOXM1, KLF4, and TCF7L2 were the most significant TFs targeting the greatest number of genes associated with HGPTs. Among 10 TFs, SIN3A and VDR showed the most interactions with intermediate proteins and kinases (Fig. 5).

### Long-non coding RNA (LncRNA) prediction

Long-non coding RNAs (LncRNAs) were shown to have crucial roles in regulating cancer migration, invasion and metastasis. lncRNAs were analyzed using the lncHUB database linked in Enrichr. We identified the top 10 lncRNAs correlated to up- and down-regulated genes (Table 2).

**Table 2:** Identification of the key lncRNAs and genes involved in ovarian cancer.

Term	*P*-value	Target genes
Key lncRNAs targeting up-regulated genes (ChEA)		
HMMR-AS1	4.08E−26	CDCA5; KIF14; MCM10; KIF11; KIF15; MELK; PRC1; DEPDC1; NUSAP1; CKS2; PBK; ECT2; CEP55
LINC01775	1.50E−23	CENPF; MELK; PRC1; CDCA5; KIF14; DEPDC1; NUSAP1; PBK; MCM10; KIF11; CEP55; KIF15
SGO1-AS1	4.48E−21	CENPF; MELK; PRC1; CDCA5; KIF14; DEPDC1; NUSAP1; MCM10; KIF11; CEP55; KIF15
RRM1-AS1	4.48E−21	CENPU; MELK; PRC1; CDCA5; DEPDC1; NUSAP1; PBK; MCM10; KIF11; CEP55; KIF15
DIAPH3-AS1	4.48E−21	CENPF; MELK; PRC1; KIF14; DEPDC1; PBK; MCM10; KIF11; ECT2; CEP55; KIF15
PRC1-AS1	1.09E−18	CENPU; MELK; PRC1; CDCA5; KIF14; NUSAP1; MCM10; KIF11; CEP55; KIF15
DEPDC1-AS1	1.09E−18	CENPU; MELK; PRC1; CDCA5; DEPDC1; NUSAP1; CKS2; PBK; KIF11; CEP55
CSRP3-AS1	2.17E−16	MELK; PRC1; CDCA5; DEPDC1; NUSAP1; KIF11; ECT2; CEP55; KIF15
APOBEC3B-AS1	3.52E−14	CENPU; MELK; PRC1; CDCA5; NUSAP1; MCM10; KIF11; CEP55
H2AZ1-DT	4.64E−12	CENPU; MELK; PRC1; CDCA5; NUSAP1; CKS2; PBK
TMPO-AS1	4.64E−12	PRC1; CDCA5; KIF14; DEPDC1; NUSAP1; KIF11; KIF15
ODF2-AS1	4.91E−10	CENPF; PRC1; KIF14; MCM10; KIF11; KIF15
POLH-AS1	4.91E−10	CENPF; KIF14; PBK; MCM10; KIF11; KIF15
CNOT10-AS1	4.91E−10	MELK; DEPDC1; PBK; MCM10; KIF11; KIF15
CDKN2A-DT	4.13E−08	CENPU; MELK; PRC1; DEPDC1; NUSAP1
Key lncRNAs targeting down-regulated genes (ChEA)		
PRICKLE2-AS1	1.30E−04	APC; LAMA4; PARVA
OBI1-AS1	1.30E−04	APC; ARHGEF7; ARHGEF6
LINC01945	1.30E−04	LAMA4; CNTN4;WNT16
KCNAB1-AS1	0.0044345	SFRP1; MGP
LRRC8C-DT	0.0044345	APC;ARHGEF6
LINC02613	0.0044345	SFRP1; MGP
LINC02150	0.0044345	APC; ARHGEF7
LINC00583	0.0044345	SFRP1; MGP
CYP1B1-AS1	0.0044345	COL8A1; PARVA
LINC01581	0.0044345	LAMA4; PARVA
ARHGEF7-IT1	0.0044345	APC; ARHGEF7
WARS2-IT1	0.0044345	APC; ARHGEF6
TEX26-AS1	0.0044345	LAMA4; COL8A1
COL4A2-AS2	0.0044345	LAMA4; PARVA
ECI2-DT	0.0044345	APC; ARHGEF6

### Exploring the protein atlas database: an in-depth analysis

**Figure 6:**
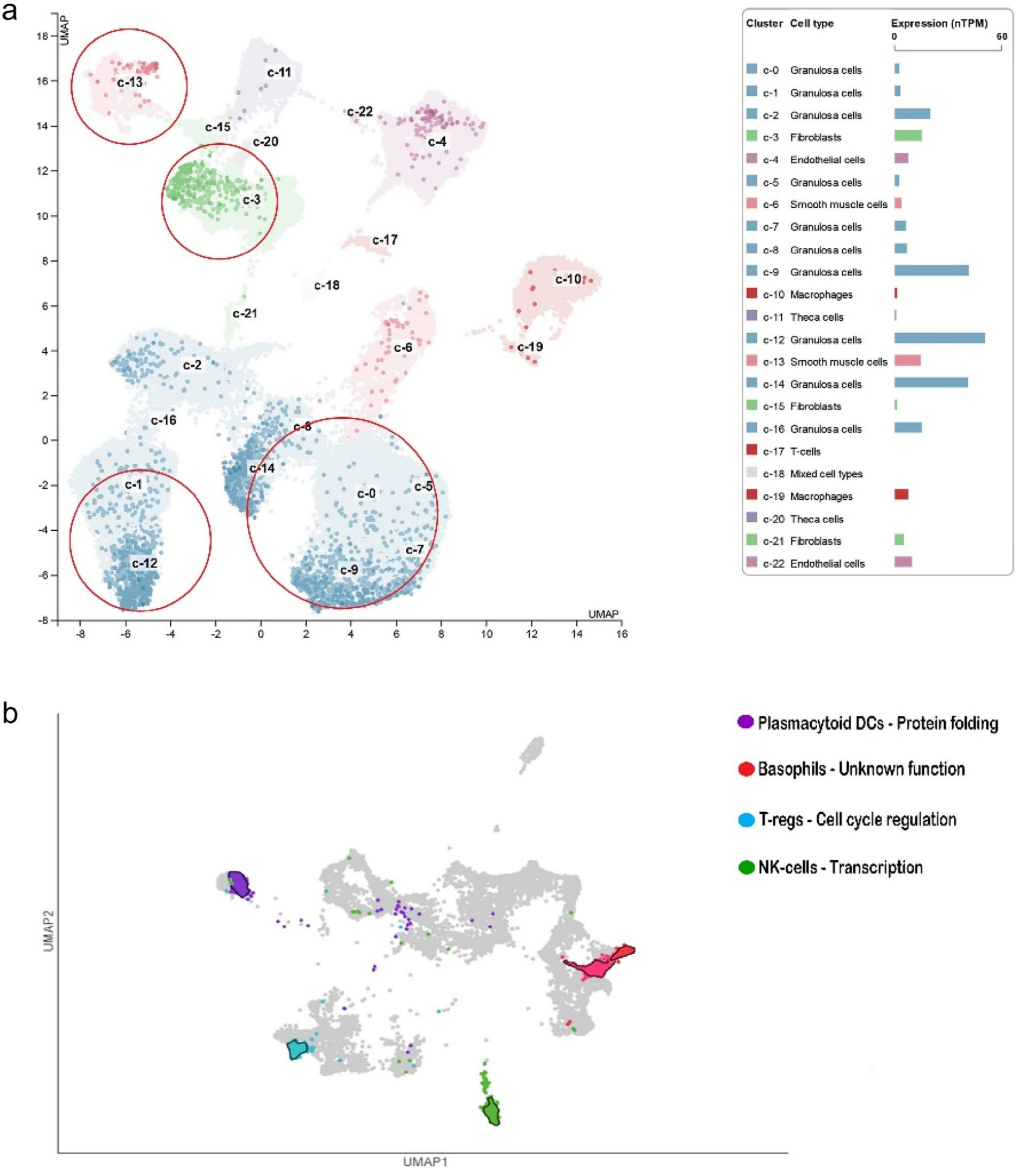
Cluster cell type analysis. (**a**) Clustering of gene markers recognized by UMAP including granulosa cells, fibroblasts, and smooth muscle cells in the cell types. (**b**) Clustering of gene markers in immune cell types.

**Figure 7:**
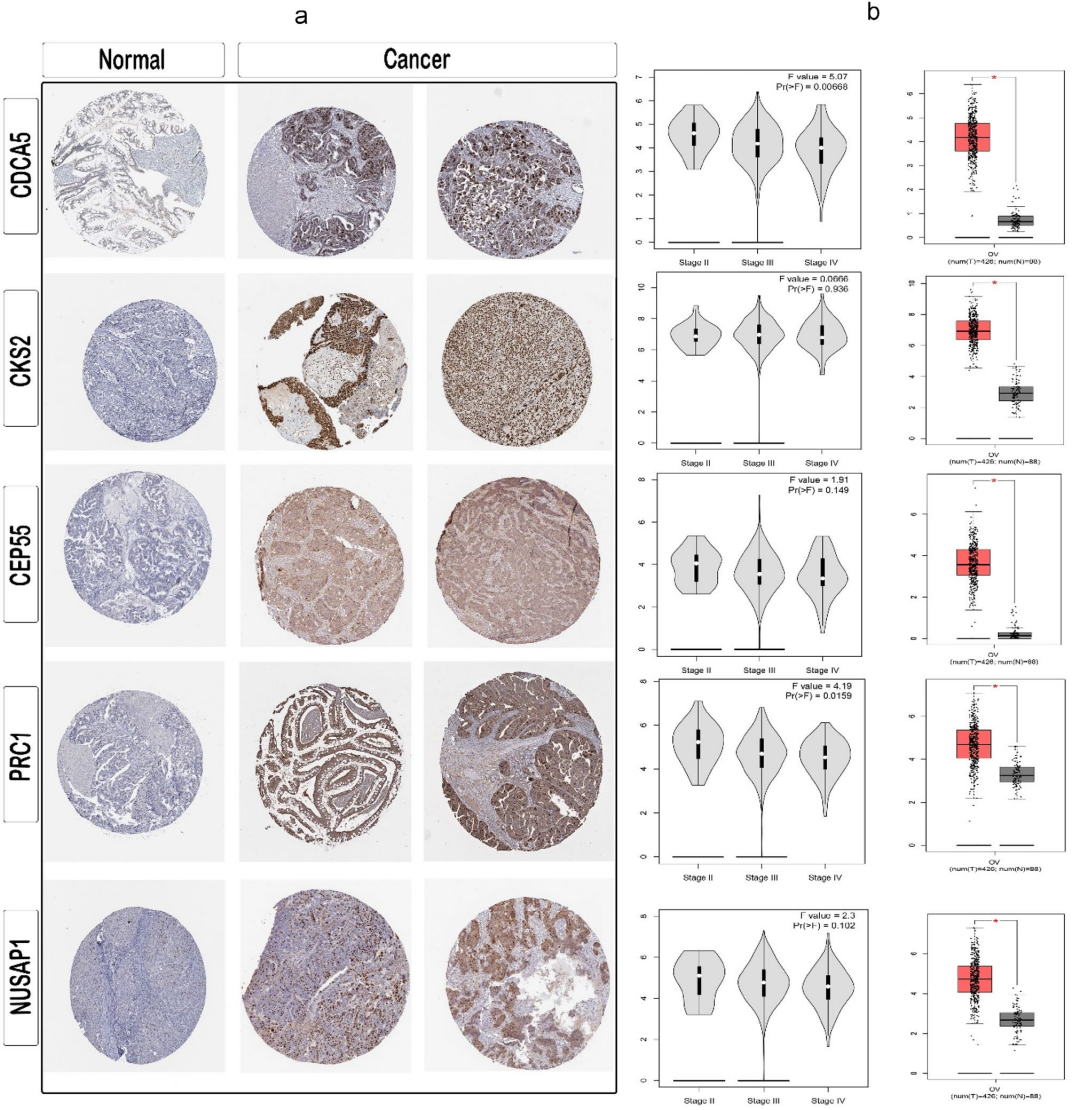
Protein expression analysis. (**a**) Analysis of five high-grade primary tumor (HGPT)-related gene markers based on the Human Protein Atlas (HPA). (**b**) The expression level of potential hub genes is based on the gene expression profiling interactive analysis (GEPIA) database.

The hub genes were chosen from the PPI network of HGPT-related genes using cytoHubba. Among the top 20 genes associated with HGPT-related genes, five hub genes, including CDCA5, CKS2, CEP55, PRC1 and NUSAP1, were evaluated in the protein atlas server. The gene information of these gene markers was first obtained from single-cell data and then clustered in OC using the UMAP plot, displaying these gene clusters in granulosa cells, fibroblasts, and smooth muscle cells (Fig. 6a). Subsequently, immune cell type section analysis showed that gene markers, such as CKS2, are clustered in plasmacytoid dendritic cells (pDCs) where they help fold proteins; PRC1 and NUSAP1, CEP55, and CDCA5 were clustered in basophils, regulatory T cells (T-regs; where it helps control the cell cycle), and natural killer cells (NK cells; where it obviously makes copies of DNA), respectively (Fig. 6b). Moreover, the GEPIA database was used to examine the expression of the candidate hub genes in HGPT-related genes. Our outcomes confirmed that the expression of potential hub genes (at the mRNA level) is much higher in HGPT samples than those in normal tissues (Fig. 7a). The information of five genes in OC were evaluated after assessing the hub genes (Fig. 7b).

### Identification of significant survival-related genes

**Figure 8:**
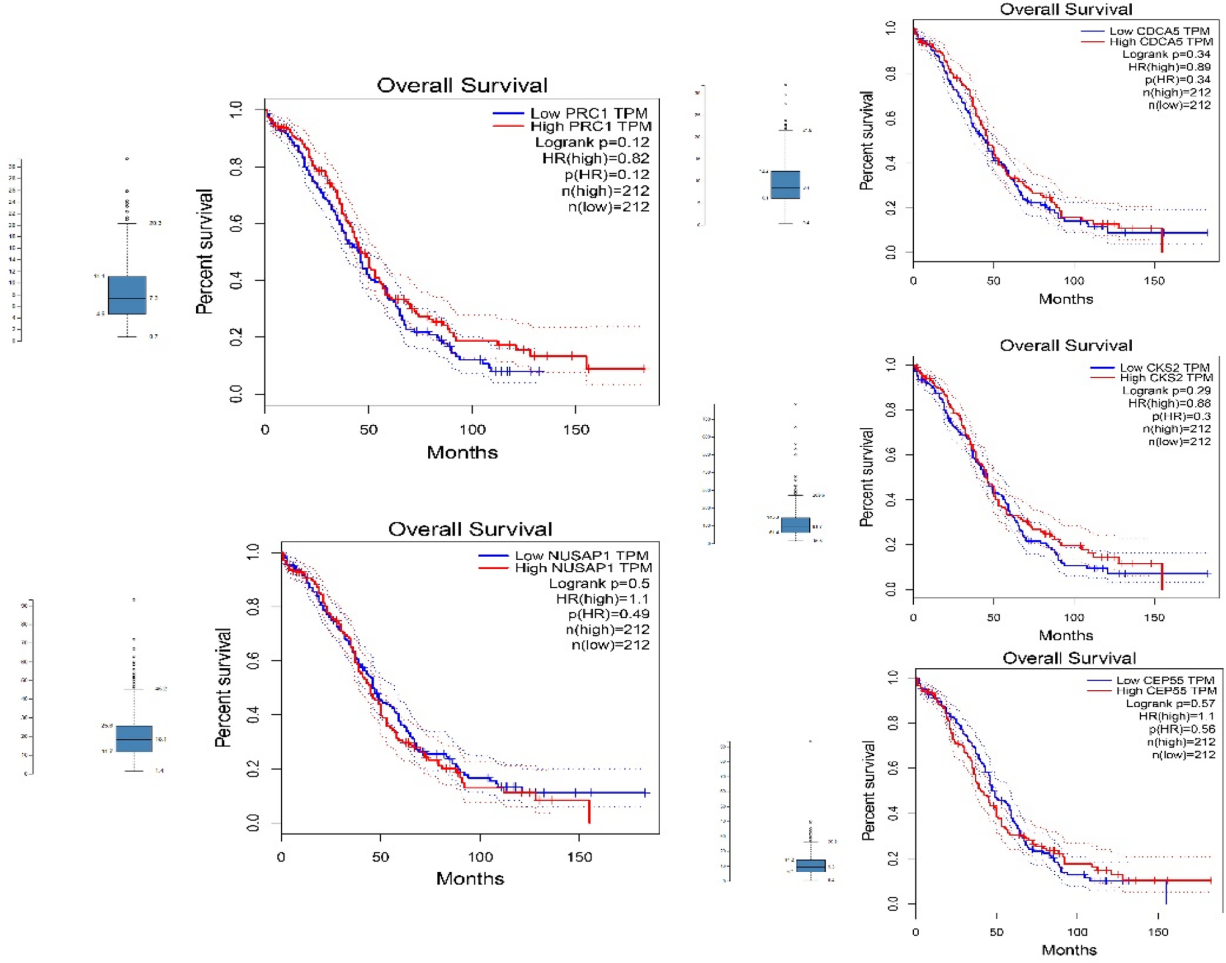
Analysis of the overall survival (OS) of five hub genes in ovarian cancer (OC) patients. The TCGA database illustrates the impact of CDCA5, CKS2, CEP55, PRC1, and NUSAP1 genes on the OS rate of patients with OC. All five graphs contain blue low and red high TPM lines, which are normalized by GAPDH.

According to the gene expression, GEPIA analyzes OS or disease-free survival (DFS, also known as relapse-free survival [RFS]). GEPIA uses the log-rank test, usually known as the Mantel-Cox test, in order to test hypotheses. Both adjustable cohort thresholds and the utilization of gene pairs are possible. It is also possible to add the cox proportional hazard ratio and the 95% confidence interval in the survival plot. We also utilized survival plots created by GEPIA to compare the expression levels of hub genes in OC and normal tissues (GEPIA). According to the Fragments Per Kilobase of transcript per Million mapped reads (FPKM) value of each gene, patients were divided into two expression groups, and the relationship between patient survival and expression levels was measured. In the OC dataset, hub genes include CDCA5, CKS2, CEP55, PRC1, and NUSAP1 with confidence intervals less than 0.05; the hazard ratio was calculated by using the Cox PH Model (Fig. 8).

### Subcellular location and immunohistochemistry functions

**Figure 9:**
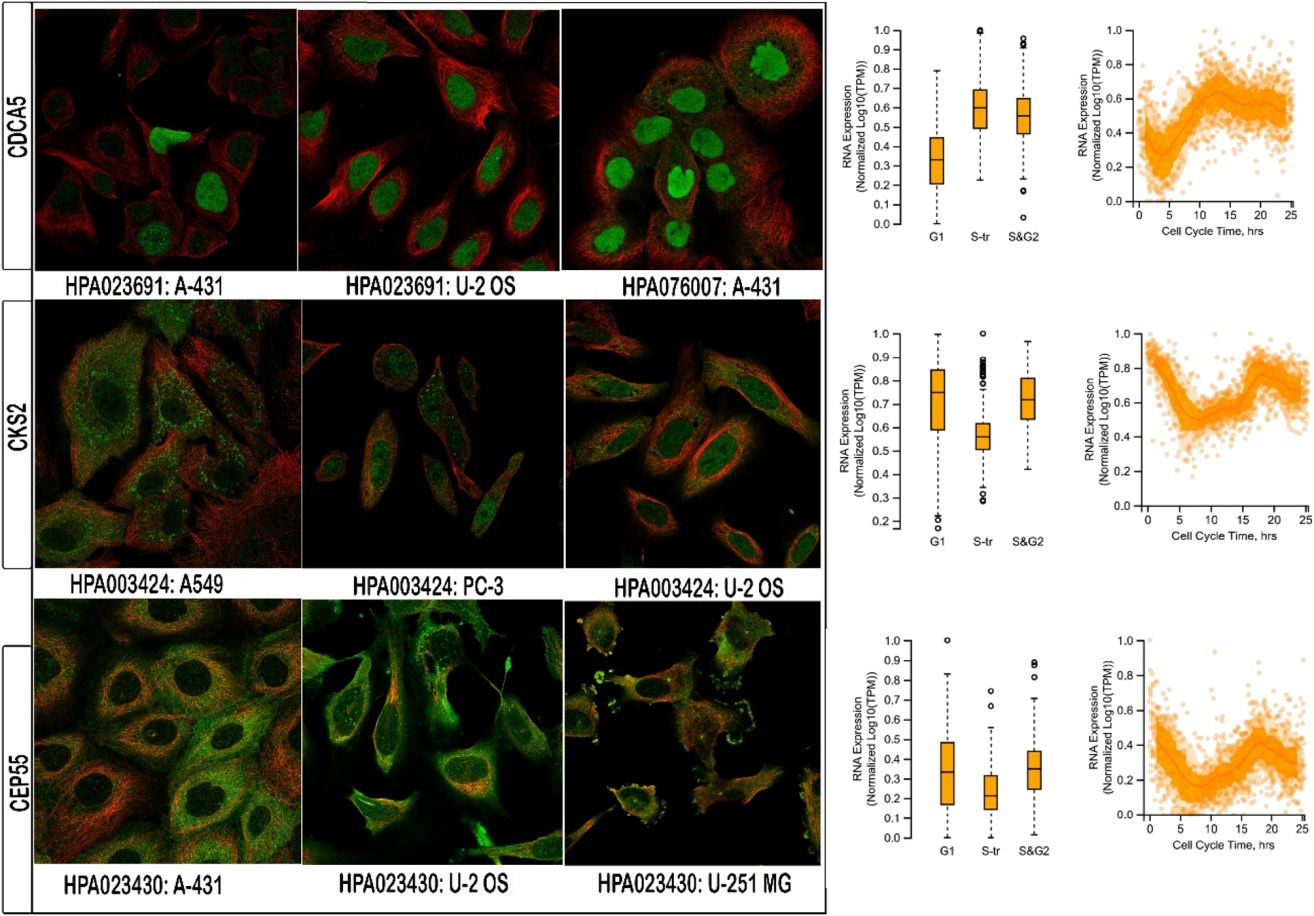
Subcellular summary. (**a**) CDCA5 was localized to the nucleoplasm where the antibodies (HPA023691 and HPA076007) were used for this analysis; CKS2 was localized to the mitochondria and cytosol where the antibody (HPA003424) was used for this analysis; and CEP55 was localized to the plasma membrane and centriolar satellite where the antibody (HPA023430) was used for this analysis. (**b**) Analysis for determining RNA expression and cell cycle phase in single cells.

The subcellular section of the database refers to high-resolution, multicolor images of labeled proteins by indirect immunocytochemistry/immunofluorescence (ICC-IF). It provides spatial analysis about protein expression patterns in order to define the subcellular localization to cellular organelles and structures at the single cell level. HPA contains images of histological sections from normal and cancer tissues, which have been obtained by immunohistochemistry. Antibodies are labeled with DAB (3,3′-diaminobenzidine) and the resulting brown staining indicates where an antibody has bound to its corresponding antigen. In this section, we found that biomarkers, including CDCA5, CKS2 and CEP55, are recognized through HPA023691 and HPA076007, HPA003424, and HPA023430 antibodies, respectively (Fig. 9).

## Discussion

OC prognosis remains challenging, primarily due to late-stage diagnosis^22^, highlighting the need for innovative therapeutic strategies to investigate the molecular intricacies underlying OC development, recurrence, and metastasis. By exploring the gene expression landscape of advanced-stage HGPTs, we aimed to uncover potential insights into these intricacies. Leveraging omics sciences, such as transcriptomics and proteomics, our analysis focused on key entities, including hub genes, TFs, miRNAs, lncRNAs, kinases and PPIs. These entities play crucial roles in HGPT-associated gene or protein expression and offer potential therapeutic targets.

Moreover, our investigation of significant co-expression genes has shed light on potential targets related to HGPT-associated hub genes. These genes, through their co-expression patterns, may serve as indicators of cancer progression or regression. Our comprehensive in silico analysis aimed to address critical questions regarding the key signaling pathways for HGPT, identify hub genes in the PPI network, uncover regulatory TFs and kinases, determine potential antibody targets for hub genes, and elucidate the roles of miRNAs and lncRNAs in the behavior of HGPT cancer cell lines.

Our GSEA analysis revealed that HGPT-related genes play a significant role in metabolic processes. The one-carbon pool by folate metabolism, in particular, emerges as a pivotal pathway with far-reaching implications. This pathway plays a critical role in various physiological processes, including biosynthesis, amino acid homeostasis, epigenetics, and redox defense. Disruptions within this pathway can fundamentally alter the course of cancer initiation and progression. Folate and choline, central components in the one-carbon metabolism, play a key role in the pathobiology of epithelial OC (EOC), underscoring the position of EOC as one of the most lethal gynecological malignancies^23^.

A nuanced understanding of the signaling intricacies in HGPTs is crucial for the development of therapeutic approaches capable of balancing efficacy, reducing toxicity, and increasing chemotherapy sensitivity^24^. In our PPI network analysis, we found a complex interplay of direct and indirect interactions among genes linked to HGPTs. The interaction density of each gene indicates its potential therapeutic value. In addition, co-expression patterns within the PPI underscore the intricate relationships between hub and non-hub genes, shedding light on potential avenues for miRNA-based therapies. We identified 10 hub genes, including KIAA0101, RAD51AP1, FAM83D, CEP55, PRC1, CKS2, CDCA5, NUSAP1, ECT2 and TRIP13. The mRNA and protein levels of hub gene expression were verified using GEPIA and HPA databases, respectively. Five genes, including CEP55, PRC1, CKS2, CDCA5 and NUSAP1, were found to be overexpressed in OC. Most importantly, several antibodies, including HPA023691 and HPA076007, HPA003424, and HPA0230, play key roles in the CDCA5, CKS2, and CEP55 genes, respectively; for example, HPA023691 is an antibody against CDCA5, a cell cycle regulatory protein with a crucial role in the development of several human malignancies^25^. Analysis of GEPIA and the Protein Atlas database demonstrated that CDCA5, CEP55, PRC1, CKS2, and NUSAP1 have the potential to serve as diagnostic and prognostic markers for HGPTs, as well as therapeutic targets for OC^26^. According to a recent study, overexpression of CEP55 has resulted in spontaneous tumorigenesis, which raises the risk of metastasis^27^. Our findings demonstrated that tumor suppressor miRNAs, such as miR-215-5p, could decrease tumor development^28^. In addition, we provided a significant list of lncRNAs for diagnosis; based on our findings, HMMR-AS1, LINC01775 and SGO1-AS1, for example, may be useful in OC diagnosis.

Recent advancements in the understanding of the fundamental molecular mechanisms underlying cancer cell signaling have revealed the pivotal role of kinases in the carcinogenesis and metastases of various cancer types^29^. Since most protein kinases, when constitutively overexpressed or active, promote cell proliferation, survival and migration, they are consequently associated with oncogenesis^30^. We could find kinases and determine their network interaction with the hub genes via X2K. At the end, the most significant kinases, including CDK1, AKT1, MAPK14, MAPK1 and CSNK2A1, were identified in this study. CDK1 is a family member of cell cycle regulatory proteins involved in cell cycle maintenance. Given that CDK1 overexpression was found to be associated with cancer, CDK1 inhibitors may restore equilibrium to the skewed cell cycle system and serve as an effective therapeutic agent^31^.

In conclusion, findings from our study shed light on the critical factors associated with the development of HGPTs, paving the way for improved therapeutic interventions. By integrating omics data, we aimed to develop novel treatment approaches for patients with OC.

### Supplementary Information

Supplementary Information.

## Data Availability

1. The datasets generated during and/or analysed during the current study are available in the [GSE DataSets and Human Protein Atlas] repository, [https://www.ncbi.nlm.nih.gov/geo/query/acc.cgi?acc=GSE18520] and [https://www.proteinatlas.org/ accession numbers, ENSG00000146670, ENSG00000138180, ENSG00000123975, ENSG00000198901 and ENSG00000137804]. 2. All data generated or analysed during this study are included in this published article (and its supplementary information files).

## Abbreviations

OC: Ovarian cancer
HGPT: High grade primary tumor
GEO: Gene expression omnibus
DEG: Differently-expressed gene
GSEA: Gene set enrichment analysis
ChEA: ChIP enrichment analysis
X2K: eXpression2Kinases
HPA: Human protein atlas
TF: Transcription factor
PPI: Protein–protein interaction
GEPIA: Gene expression profiling interactive analysis
